# Analytical Review of the Current State of Technology, Structure Formation, and Properties of Variatropic Centrifugally Compacted Concrete

**DOI:** 10.3390/ma17081889

**Published:** 2024-04-19

**Authors:** Evgenii M. Shcherban’, Sergey A. Stel’makh, Levon R. Mailyan, Alexey N. Beskopylny, Alla S. Smolyanichenko, Andrei Chernil’nik, Diana Elshaeva, Nikita Beskopylny

**Affiliations:** 1 Department of Engineering Geology, Bases, and Foundations, Don State Technical University, 344003 Rostov-on-Don, Russia; au-geen@mail.ru; 2 Department of Unique Buildings and Constructions Engineering, Don State Technical University, 344003 Rostov-on-Don, Russia; sergej.stelmax@mail.ru (S.A.S.); lrm@aaanet.ru (L.R.M.); chernila_a@mail.ru (A.C.); diana.elshaeva@yandex.ru (D.E.); 3 Department of Transport Systems, Faculty of Roads and Transport Systems, Don State Technical University, 344003 Rostov-on-Don, Russia; 4 Department of Water Supply and Sewerage, Don State Technical University, 344003 Rostov-on-Don, Russia; arpis-2006@mail.ru; 5 Department Hardware and Software Engineering, Don State Technical University, 344003 Rostov-on-Don, Russia; beskna@yandex.ru

**Keywords:** centrifugally compacted concrete, centrifugation, concrete, variatropy, vibrocentrifugation

## Abstract

Current regulatory documents and the scientific literature lack a theoretical framework and practical guidance for calculating centrifugally compacted reinforced concrete structures, taking into account the variatropy of their structure and the material’s characteristics across the section. A problem related to this research lies in the need to form a systematized, theoretical, and practical knowledge base about variatropic concretes, the importance of which has been proven by various scientists without, to date, the creation of a unified scientific methodological base. The importance of this study is linked to the need for the world’s construction projects and processes to transition to the most economically, materially, and resource-efficient types of building structures, which, of course, include structures made of variable-type concrete. This study’s objective is to fill these scientific and engineering gaps. The purpose of this study was to systematize the existing knowledge base about the technology, structure formation, and properties of variatropic concrete, using an analytical review of previously conducted studies by ourselves and others, both in Russia and abroad. A theoretical justification for the formation of the structure of variatropic materials is presented. An analysis of the basic physical and mechanical properties of variatropic concretes is carried out and the features of their microstructures are considered. The main structures created using centrifugation technology are considered. Variatropic concrete has an increased amount of mechanical characteristics compared to traditional concrete, on average by up to 45%. The durability of variatropic concrete is improved, on average, by up to 30% compared to conventional concrete.

## 1. Introduction—General Picture of the Current State of Variatropic Concrete

The relevance of the ongoing research is justified by the need to systematize the existing knowledge and scientific information about the technologies, structure formation, and properties of variatropic concrete, obtained due to the physical effect of centrifugal compaction. The importance of such systematization is due to the advantages of such concretes over analogues with a simple structure, which makes it possible to achieve design, technological, calculation, and production advantages.

In this regard, this study is divided into three semantic blocks, relating, first, to technological tasks and issues of the equipment. Second are the issues of structure formation, the processes of variatropic concrete, depending on formulation and technological factors and the nature of the physical and chemical processes of structure formation. And third are the properties and characteristics of the resulting concrete, both from the point of view of physical mechanics and from the point of view of operational reliability in actual building structures.

Concrete and several kinds of reinforced concrete structures rightfully play the major role in modern construction. The active development of the construction industry in recent years is not only inextricably linked with the growing needs for buildings and structures, but also implies a constant increase in the requirements for their safety and functionality [1,2,3]. This problem opens up significant prospects for researchers, scientists, and engineers to find solutions. These decisions relate to new technologies for creating buildings and structures, which make it possible to achieve a new quality level, even for objects of enhanced responsibility, through the application of new technological developments; expensive, complex equipment; and software systems [4,5,6]. However, on the other hand, an approach related to the search for hidden reserves and unexplored areas in already known types of building structures, which, for certain reasons, were not taken into account in calculations and design, may also be of interest. Essentially, this way of solving the problem concerns either a new approach to already known building structures, proven over decades of practical application and discussed in detail in regulatory documents, which implies the use of new materials, or a certain improvement in the technology for implementing design solutions, considering modern development prospects in the field of construction technologies, structures, calculations, and design [7,8,9,10].

Such technologies include the production of concrete using centrifugal compaction. In accordance with this, when creating reinforced concrete structures, centrifugal force acts on the concrete mixture, which leads to the formation of a heterogeneous structure, the peculiarity of which is an increase in density and strength in relation to the distance from the axis of symmetry of the element. Such structures are called variatropic and have been studied for various concrete mixture compositions [11,12,13,14,15,16,17,18,19]. As a rule, ring-section racks are manufactured using centrifugal compaction technology. Such elements are in demand in many areas, such as, for example, industrial and hydraulic engineering, as well as in the fields of energy and transport. The reinforcement of such samples is carried out using longitudinal reinforcement, evenly distributed along the perimeter of the cross-section, and connected by transverse spiral or ring reinforcement. Columns made in this way are equally resistant to mechanical stress in all directions, which makes them optimal and economical, especially under significant horizontal loads [20].

**Figure 1:**
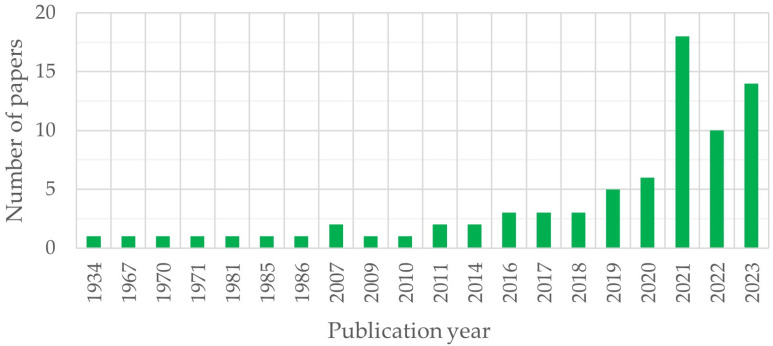
Main articles reviewed in this study.

Centrifugation technology, as a means of compaction, found its application at the beginning of the 20th century in metallurgy. At the same time, it began to be used for molding reinforced concrete supports, pipes, and other elements of annular cross-section. The basic theoretical principles of centrifugation technology were first formulated by E. Marquardt [21], based on the ideas of the science of concrete, in the 1920s–1930s. He used some theoretical principles from solid mechanics and obtained dependencies that are still used today in the production of centrifuged concrete and reinforced concrete elements. Since concrete is an elastic–plastic–viscous material, the laws describing the physical and mechanical properties of solids are not applicable to it, since the compressive deformations of a concrete mixture under pressure develop gradually, while the force applied to a solid almost immediately causes a corresponding deformation. In this regard, it became necessary to reconsider several technological issues related to new ideas about the structure of cement paste and its rheological properties. The main articles reviewed in this review, by year of publication, are shown in Figure 1.

This comprehensive review analyzes the physical and mechanical properties of variatropic concrete, while also critically evaluating formulation techniques and input/output variables. Additionally, the focus is on recent research concerning the microstructural properties and durability of variatropic concretes. The properties of variatropic concrete at all hardening stages are influenced by certain factors.

The scientific novelty of this research lies in creating a new system of knowledge, combined according to technological, structural, and characteristic criteria, reflecting the knowledge base in these areas that exists in the domestic and foreign scientific base.

This study is intended to tackle problems related to systematizing existing ideas and to build a solid, empirical, and literary groundwork for advancing the world theory of variatropic concrete. When conducting a literature review and scientific background analysis, it is important to understand the specific challenges that need to be addressed when implementing centrifugal reinforced concrete construction (RCC) features. Such problems include, firstly, the weak degree of regulation, the features of centrifugal RCCs, and the differences in their characteristics when calculating structures. Secondly, the technological process of obtaining centrifugal RCCs with specified characteristics is difficult to control. Thirdly, the scientific and practical base of previous studies on the implementation of the characteristics of centrifugal RCCs is poorly generalized to date.

The purpose of this study is a detailed, in-depth literature review and a thorough analysis of the current state, assessment of prospects, and identification of specific vectors for the development of the market for variatropic concrete in global construction and production. To achieve this goal, we have solved the following tasks:

(1) a review was carried out on a wide range of world research, based on the best existing world practices and scientific achievements in the field of variatropic concrete;
(2) the major formulation, constructive, technological, engineering, and scientific approaches to solving the problems of the best and most environmentally and economically efficient variatropic concretes for a wide variety of types of climatic zones, regions, buildings, structures, and various levels of their responsibility were identified;
(3) the key factors and major criteria influencing the properties and efficiency of variatropic concrete were identified;
(4) the main fundamental relationships between the composition, structure, and properties of variatropic concrete were determined;
(5) the relationship was identified at both the micro and macro levels in the structure’s formation and properties of variatropic concrete;
(6) the performance of variatropic concrete in structures was assessed and the main areas of use of such structures were identified;
(7) a generalized analysis and discussion of the current state, assessment of prospects, and identification of specific vectors for the development of the market for variatropic concrete in global construction and production was carried out.

The significance of this review lies in the analysis and systematization of new scientific research, the planning of future experiments for scientists involved in variatropic concrete, and, from a practical point of view, in the applicability of the received recommendations and knowledge for the applied industry of production of building products and structures in terms of reinforced concrete elements with a variatropic concrete structure.

## 2. Types of Centrifugal Machines for Producing Variatropic Concrete

Depending on the method of securing the forms and bringing them into rotation, three types of centrifugal machines are distinguished, as follows: the first type—axial or spindle—in which the form is firmly clamped at the ends between two headstocks, with face washers centrally mounted on rotating spindles; the second type—free-roller— in which the form freely rests with its bandages on rotating rollers and is pressed against them under the influence of its weight or the pressure of the upper rollers, the transmission of rotation from the drive roller to the mold with the concrete mixture is carried out via frictional engagement; and the third type—gyroscopic—in which the form is freely suspended on belts and is driven into rotation due to the friction forces between the text rope belts and the flanges of the form [22,23,24,25].

**Figure 2:**
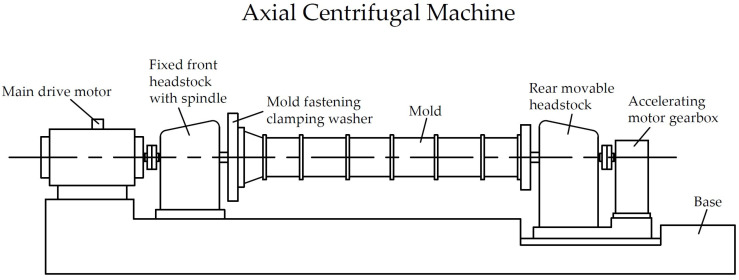
Diagram of an axial centrifugal machine.

An axial (spindle) centrifugal machine (Figure 2) has a fixed front headstock and a movable rear headstock, with clamping faceplates to secure the mold at the ends. The spindles of the headstock and tailstock are driven into rotation by the main, and accelerating, electric motors. The main motor operates on a direct current (through a rectifier) and can smoothly change the speed of the form within a fairly wide range, from approximately 60 to 1000 rpm, depending on the resistance introduced into the electrical circuit. The accelerating motor operates on a conventional alternating current and is turned on only during the startup and acceleration of the centrifuge.

Axial centrifuges allow for high rotation speeds of the molds with the concrete mixture and also for a better compaction. The main disadvantage of this method is the complexity of its design and the difficulty of loading the concrete mixture into a mold mounted on a centrifuge. Usually, in these cases, the mixture is loaded into the half-mold, away from the centrifuge, from a self-propelled concrete paver after installing the reinforcement cage [22,23,24,25].

**Figure 3:**
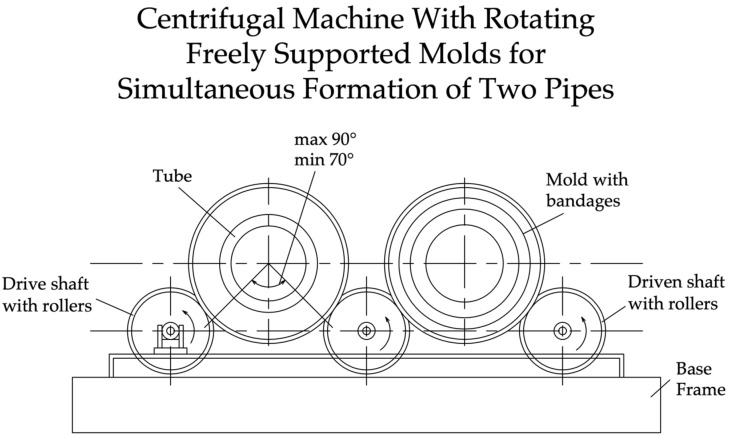
Scheme of a free-roller centrifuge.

A free-roller centrifuge (Figure 3) is simpler in design than an axial centrifuge and has a lower installed engine power.

The centrifuge consists of a foundation frame and two or three parallel horizontal shafts with rollers mounted on them at the ends. The number of rollers depends on whether one or two pipes are being formed at the same time. The form rests on the rollers with its bandages and, because of significant frictional forces (with the large weight of the form with the concrete mixture), is driven into rotation by the rollers of the drive shaft; the remaining rollers, being engaged with the mold bandages, are also driven into rotation. This allows the use of roller centrifugal machines that simultaneously form several pipes with one drive and a corresponding number of driven shafts with rollers. The disadvantage of free-roller centrifuges is the significant amount of noise they make during their operation, which adversely affects the health of workers in the workshop. This noise is created by metal rollers, on which mold bands roll at high speed. Significant centrifugal accelerations developed when the molds with concrete mixture are insufficiently balanced and at high rotation speeds can lead to the molds being thrown off the machine. Therefore, when operating free-roller centrifuges, reliable protective guards are required, as well as periodic checking of the balance of the forms [22,23,24,25].

**Figure 4:**
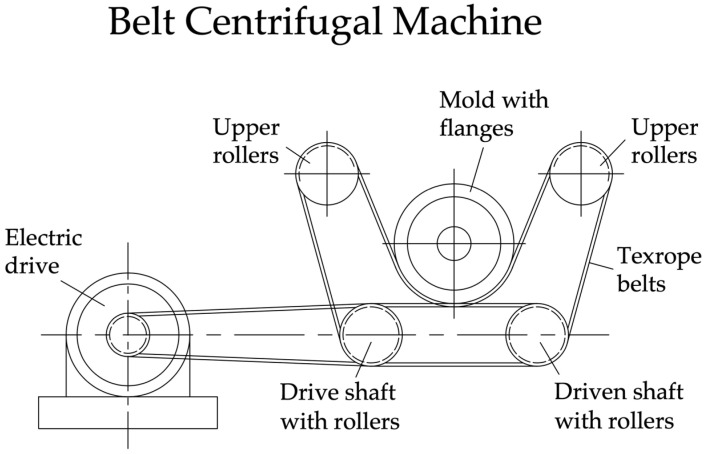
Belt centrifuge diagram.

A belt (gyroscopic) centrifuge (Figure 4) comprises four longitudinal shafts, of which one drive shaft is connected by a drive to an electric motor. The remaining shafts are driven; rollers with ring grooves for texrope belts are evenly placed along the length of the shafts.

The rollers on the shafts, together with the belts put on them, form a series of parallel supports (from 4 to 6, depending on the length of the molded pipes), on which the metal mold rests with its flanges. Texrope belts (28 in total) are placed and tensioned on the rollers in such a way (Figure 4) that they form a U-shaped bed for the mold. The rotation of the mold at high speed on these machines occurs smoothly and silently. Circular grooves on the flanges of the mold create unique rolling paths for the mold. The belt centrifuge eliminates vibration and there is no danger of the mold being thrown out of the installation, which allows rotation speeds of up to 1500 rpm. The main disadvantage of belt centrifuges is the relatively short service life of texrope belts, due to their stretching [22,23,24,25].

In general, each of the considered types of centrifuges has one disadvantage or another. The choice of installation for producing variatropic concrete will depend on many factors, the main ones of which are the purpose and volume of the production of variatropic concrete; the parameters of the room in which the installation is located; and the necessary rotation parameters of the installation to obtain the required variatropic concrete.

## 3. Theoretical Rationale for the Formation of a Variatropic Structure of Centrifuged Concrete

The essence of the centrifugal molding method is that the mold, together with the concrete mixture loaded into it, rotates around its longitudinal axis at a given speed. Under the influence of developing centrifugal forces, the particles of the mixture are thrown towards the walls of the mold, pressed against them, and the mixture is distributed in the mold in a layer of uniform thickness. The resulting pressing pressure, the magnitude of which is proportional to the mass of the particles of the concrete mixture; their distance from the axis of rotation; and the square of the angular velocity help to squeeze out part of the mixing water, which leads to the compaction of the concrete [23,24].

Let us consider a detailed explanation of the principles of the laws of formation of the structure of centrifugal concrete. The main principle of the law of the centrifugal formation of concrete structures is based on the process of the physical laws related to centrifugal force and angular velocity. In the case of the manufacture of centrifugal reinforced concrete (RC), the nature of the formation of the concrete structure, being a technological task, directly depends on the fundamental physical parameters of the centrifugal compaction–centrifugal force and angular velocity. Important parameters also include the radius of rotation, as well as the density and mass of the centrifuged concrete mixture. In this regard, the basic principles of concrete structure formation, according to this law, must be maintaining the constancy of these parameters, so that the formulas of fundamental physics can be applied in every repeating experiment or technological production. The third principle of centrifugal concrete production is the direct relationship between the compositions, structure, technology, and properties of the forming concrete, which allows methods of mathematical experimental planning to determine the output parameters in the form of structure characteristics and properties of centrifugal concrete by changing the input factors—rotation parameters, centrifugal force, mass changes, or the density of the concrete mixture.

As a result, products are formed that are, in cross-section, bodies of rotation with an internal cylindrical cavity and this allows for the formation of pipes and hollow tubular structures without the use of complex double molds with an internal core. The external outline of the cross-section of molded products can be different, i.e., round, polygonal, square, and others. The dimensions of centrifugally molded products can be quite large in dimension (load-bearing columns, lighting masts, transmission line supports, and connections with a length of 15 m or more) and in cross-section (pipes with a diameter of 2–2.5 m or more).

The centrifugal molding process can be divided into the following two stages: the distribution of the concrete mixture along the walls of the mold and the formation of an internal cavity in the product is the initial stage of molding the products; the final stage is the compaction of the concrete mixture in the molded product [23]. In the first stage, the plastic–concrete mixture is loaded into a rotating mold and, due to the friction against its walls, begins to rotate with it. Thanks to its plasticity and necessary mobility, the mixture is distributed in an even layer over the walls of the mold, maintaining the continuity of its structure. When the mold rotates, the particles of the concrete mixture are simultaneously exposed to two force fields—gravity and centrifugal forces [24].

The result of these forces at any point on the circle with a particle mass m = 1 can be described using Formula (1).

(1) $$\rho =\sqrt{{\left(r\omega^{2}\right)}^{2}+g^{2}-2r\omega^{2}g\cos\left(\alpha \right)}$$

At the lowest point of the mold at cos (180°) = −1, this resultant force will be equal to $r\omega^{2}+g$, that is, the particle pressed against the wall of the mold experiences a total pressure from its own weight and from the centrifugal force. At the highest point of the form, the total pressure on a particle with *m* = 1 is equal to the difference between these two forces, $r\omega^{2}-g$.

**Figure 5:**
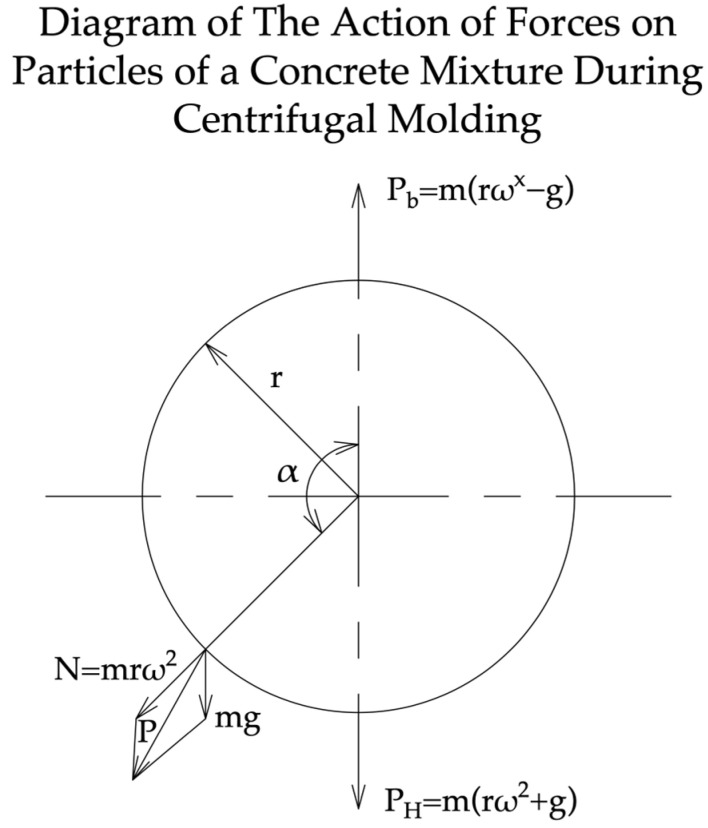
Diagram of the action of centrifugal forces on particles of a concrete mixture during centrifugal molding.

In order for the particles of the mixture, raised when the mold is rotated to the highest position, not to come off from its wall and not to fall, the forces of centrifugal acceleration and gravity acceleration must be equal. This equality can be achieved with the corresponding minimum value of the mold rotation speed, $\omega =\sqrt{\frac{g}{r}}$. Substituting the number of revolutions in minutes, instead of the angular velocity value, we obtain the minimum required number of mold revolutions at the first stage of molding, $n=\frac{300}{\sqrt{r}}$ rpm, where *r* is the internal radius of the mold in centimeters. A diagram of the action of centrifugal forces on particles of a concrete mixture during centrifugal molding is shown in Figure 5.

The actual critical number of rotations of the mold should be 1.5–2 times greater than the theoretical one, taking into account the possible vibration and shock of the mold during rotation. For medium-diameter pipes, the initial operating rotation speed during the first stage of molding ranges, on average, from 60 to 150 rpm, depending on the diameter of the molded pipe. During the compaction stage, the rotation speed of the mold increases significantly; it should be approximately 400–900 rpm, depending on the diameter of the mold and the required concrete compaction. Since plastic–concrete mixtures with a sufficient content of cement paste are used for centrifugal molding, the pressing pressure on the concrete is transmitted and evenly distributed through layers of cement paste, filling all intergranular spaces in the concrete. Under the influence of this pressure, a certain amount of free water is squeezed out of the pore cells and capillaries, and the dough, decreasing in volume, helps to bring the particles of the solid phase closer together, resulting in a decrease in the total volume of concrete and its compaction. However, the process of squeezing water out of the cement paste gradually stops, since, with an increase in the density of concrete and with the narrowing of the channels, the resistance to the movement of water in the cement paste increases until this movement completely stops (when an equilibrium occurs between the forces of hydrostatic pressure and the resistance to water movement) [23,24].

The required number of rotations of the mold during the stage of concrete compaction can be determined based on the accepted value of the pressing pressure per 1 cm^2^ of the outer cylindrical surface, according to the following formula:

(2) $$N=\frac{\rho \omega^{2}\left(R^{3}-r^{3}\right)}{3gR}$$

*ρ* is the density of the concrete mixture; *R* and *r* are the external and internal diameter of the molded tubular product; *ω* is the angular velocity; and *g* is the acceleration of gravity.

In general, the compaction process in centrifugal molding is similar to the compaction process in vibro-compression. However, during vibro-compression, due to the lower initial water content of the mixture (lower than the water–cement ratio, W/C), the value of the pressing pressure is taken to be significantly higher and the amount of squeezed water, in relation to its initial content, is less than with centrifugal molding. The centrifugal molding mode is determined by the speed of the rotation of the mold and the duration of the centrifugation process. Both of these factors, within certain limits, can be considered interchangeable [24]. To establish the optimal mode of centrifugal molding during its second stage, it is necessary to know what factors affect, and how to determine, the amount of squeezed water and the speed at which it is squeezed out of the concrete mixture, since the former determines the final density of the centrifuged concrete and the latter determines the duration of the molding cycle.

The amount of squeezed water, all other things being equal, depends on the initial water content (more precisely, the W/C value) of the mixture and the normal density of the cement. Since the pressing pressure of centrifugal forces is distributed unevenly across the thickness of the concrete wall of the molded product, the squeezing out of water in all layers of the concrete element also occurs unevenly. First, “water is squeezed out more completely from the outer layers of the product, where the greatest pressing pressure is applied; as it approaches the inner surface, the water will be squeezed out in ever smaller quantities” [23]; only as the process develops over time is a more or less uniform extraction of water from concrete and its compaction achieved over the entire cross-section of the product. All this leads to the need to lengthen the centrifugal molding process to a greater extent, to improve the thickness of the product. The duration of the process at the compaction stage ranges from 10–12 to 20–25 min for internal pipe diameters from 500 to 1000 mm, which is approximately 2–2.5 min for each cm of pipe wall thickness (in general, molding takes anywhere from 25 to 50 min). The duration of this process is a disadvantage of the centrifugal method [23,24].

The main factor that reduces the duration of the process is the magnitude of the pressing pressure of centrifugal forces or the rotation speed of the mold. It should be noted that the usually accepted value of the specific pressing pressure on concrete during centrifugal molding is approximately at the level of 0.06…0.08 MPa (at practically accepted mold rotation speeds). This value is still insufficient for the quick and effective compaction of centrifuged concrete. An increase in the efficiency of centrifugal molding should be sought in a combination of pressing by centrifugal forces with any additional pressure on the concrete mixture and with the vibration effects on it. Such technologies include the production of concrete using vibration centrifugation. In accordance with this, when creating reinforced concrete structures, the concrete mixture is simultaneously affected by vibration and centrifugal force, “which leads to the formation of a heterogeneous structure, the peculiarity of which is an increase in density and strength with distance from the axis of symmetry of the element” [16,17,25,26,27,28]. These structures were called improved variatropic structures and were investigated with different compositions of the concrete mixture. Racks made from the vibration centrifugation of reinforced concrete with an annular cross-section exhibit notable attributes, such as exceptional compressive and tensile strength, stability, and elasticity. Centrifugal machines with a combined force effect on the concrete mixture being compacted are beginning to be increasingly used in practice.

Under favorable conditions for the centrifugal compaction of concrete, the amount of squeezed water can reach 20–25% of its initial content, which, with appropriate replacement of the vacated space with solid particles, contributes to a significant increase in the density and strength of concrete (the amount of residual W/C decreases by 20–25%). The density of centrifuged concrete depends not only on the amount of water squeezed out of the concrete mixture, but also on the structure of the compacted concrete created. To avoid the harmful effects of directed steam channels on the permeability of concrete pipes, I.N. Akhverdov [23] recommended molding the pipes by feeding the mixture into the mold in 2–3 steps and compacting each layer separately; in this case, the pore channels in the body of the concrete pipe are reliably blocked and the water resistance of the pipes noticeably increases. However, such multilayer molding increases cycle time and reduces plant productivity [29].

Centrifugal placement of a concrete mixture consisting of particles of different masses is accompanied by some stratification of the mixture, with larger aggregate grains tending to be located closer to the outer layers of the concrete body and smaller, thin ones closer to the inner layers. To reduce delamination, plastic mixtures with increased viscosity should be used with a higher content of binder or finely ground additives. It is also necessary to limit the maximum size of gravel or crushed stone in concrete to no larger than 15–20 mm. Finally, during the first stage of molding, the rotation speed of the molds should not be increased unnecessarily [23,30,31].

## 4. Main Performance and Defect Characteristics of Variatropic Concrete

### 4.1. Properties of Variatropic Concrete

The key feature that distinguishes variatropic concretes is the heterogeneity of their structure, which becomes denser in the cross-section as it moves away from the longitudinal axis of the element. Along with the compaction of the structure, the physical and mechanical properties also vary [32]. The compressive strength of centrifuged annular reinforced concrete elements is more influenced by the concrete’s compressive strength compared to the square or I-section elements. The positioning of the neutral axis determines the reinforcement intensity in the tension and compression regions. Simultaneously, using high-strength longitudinal reinforcement is more effective in preventing concrete deformation on the descending branch of the stress–strain curve. Compressed and tensile reinforcement are typically found farthest from the neutral axis in other section shapes. However, accurately determining the actual strength of centrifuged concrete remains challenging in both prestressed and conventional ring-section components [33]. The test results of cubes, prisms, cylinders, or ring-section samples may not accurately reflect the physical and mechanical properties of concrete in reinforced concrete products. The actual strength of centrifuged concrete is determined by the strength of cylindrical samples. At the same time, the process of manufacturing and testing prototypes of annular cross-section is complex and determining the strength, in this case, is problematic, even for laboratory samples, not to mention actual structures. Table 1 shows the main directions of research into the properties of variatropic concretes.

**Figure 6:**
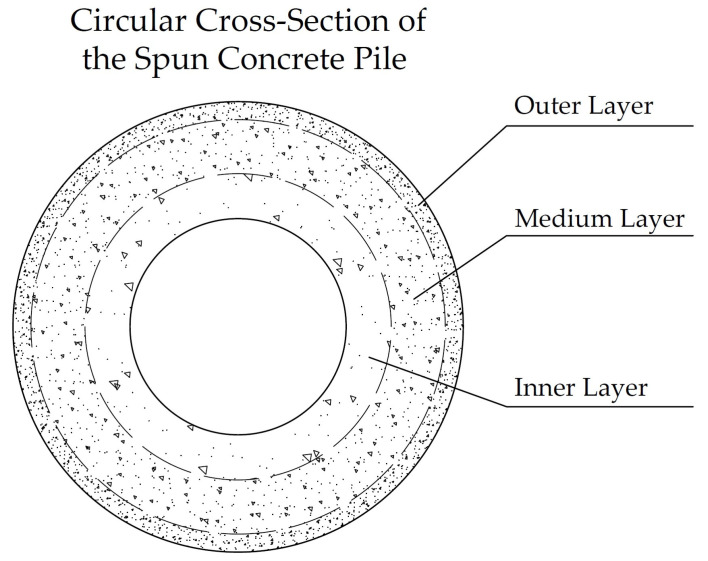
Structure of a centrifuged concrete ring element.

During the centrifugation process, a concrete structure is formed that is fundamentally different from concrete compacted only by vibration. “Centrifuged concrete, as a rule, has high tensile strength, and the compressive strength of such concrete is usually two or more times higher than that of vibrated concrete” [73]. However, the centrifugation process leads to variation in the structure of the material in the cross-section and a corresponding anisotropy of strength characteristics. The structural arrangement of centrifuged annular elements is greatly influenced by the wall thickness of the annular element. At small wall thicknesses, the aggregates of the concrete mixture are distributed in it depending on the mass of the particles. Thus, the coarse aggregate is collected closer to the external part of the element, while smaller particles are located between the granules of the coarse aggregate. Closer to the inner surface of the wall of the ring element, the concentration of coarse aggregate becomes smaller and the fine aggregate is evenly distributed in the concrete matrix. The inner layer of the ring element, as a rule, consists of cement paste, fine aggregate particles, and fine fractions. A diagram of the structure of centrifuged concrete is shown in Figure 6.

When studying the physical and mechanical properties of concrete with variable grain size, a number of important factors were noted regarding the influence of different particle size distributions on the characteristics of concrete. In particular, centrifuged concrete has a specific property, i.e., it is thin-walled, which implies the inappropriateness of using crushed stone grains of fractions larger than fraction 5–20. That is, the maximum grain size used in centrifuged concrete should be no more than 20 mm. Secondly, it is necessary to take into account not only the largest grain size, but also the ratio of grains inside the crushed stone used. That is, it is important to understand how dense the packing of particles will occur during the formation of the structure of centrifuged concrete. In particular, it is important that smaller grains of crushed stone create a dense packing between larger grains of crushed stone, while sand grains coated with cement mortar penetrate into the cavities between the forming grain conglomerates. That is, the main factors of the granulometric composition, when determining the effect on the characteristics of concrete, are the maximum grain size of the aggregate and the rational ratio between sand grains and fine and coarse fractions of crushed stone.

Through the technological process of concrete centrifugation, water in the mixture is filtered from the inner edge of the ring element to the outer one. This results in the formation of radially located capillaries in the cross-section of the concrete matrix. Through their combination, macro capillaries are formed within the inner section of the ring element’s wall, leading to a weakening of the concrete’s structural integrity. The concentration and overall magnitude of capillaries are determined using the initial water–cement ratio in the concrete mixture. When the optimal time and speed of centrifugation are achieved, “the residual water-cement ratio will be about 0.3” [74]. This reinforcement plays a crucial role in the compaction and hardening process of centrifuged concrete. Additionally, it determines the extent of shrinkage stresses, the occurrence of additional concrete deformations caused by shrinkage, the redistribution of stresses between concrete and reinforcement, and the stress–strain condition of concrete with transverse reinforcement [75].

The above is due to the fact that, in centrifuged elements of an annular section on the outer surface, the concrete has high strength, which decreases in the cross-section when approaching the inner surface of the element, where the strength of the concrete is the lowest. The difference in concrete strength at the outer and inner edges of an annular element can reach up to 20%, with a wall thickness of up to 71 mm. Such variation in the structure and density of concrete in the wall of a centrifuged element entails a corresponding change not only in strength and deformability, but also in all other physical and mechanical properties of the material [11,12,18].

**Figure 7:**
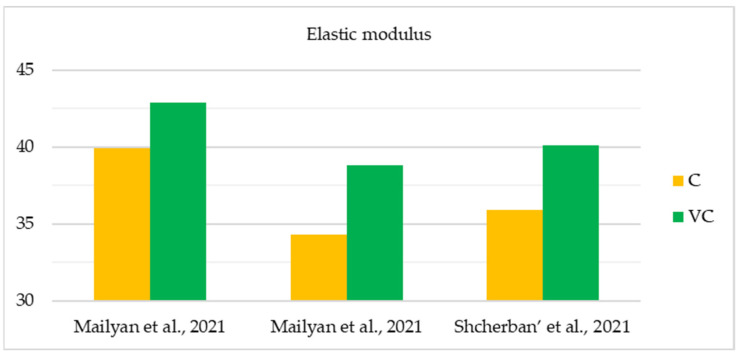
Values of the elastic modulus of concrete, depending on the manufacturing method. C—centrifuged concrete; VC—vibrocentrifuged concrete [15,16,26].

**Figure 8:**
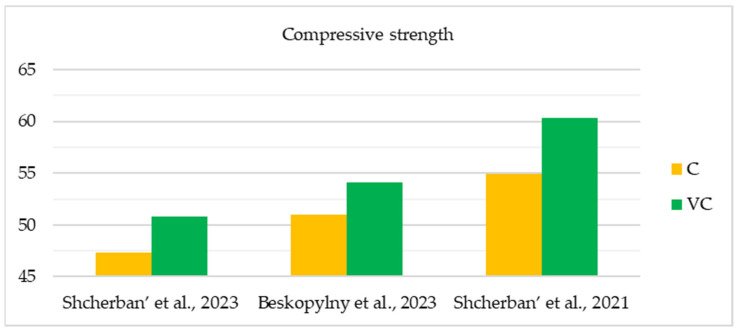
Values of the compressive strength of concrete, depending on the manufacturing method. C—centrifuged concrete; VC—vibrocentrifuged concrete [12,13,26].

**Figure 9:**
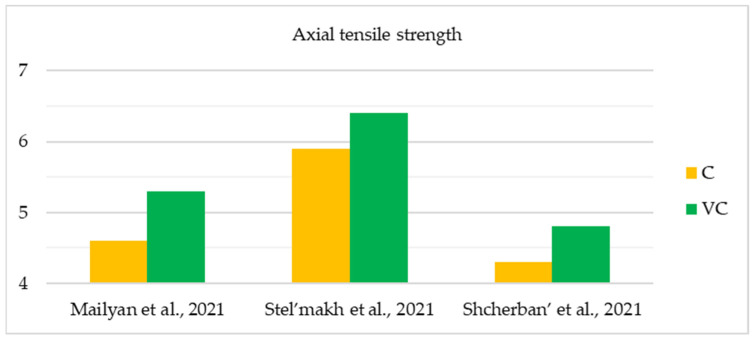
Values of the axial tensile strength of concrete, depending on the manufacturing method. C—centrifuged concrete; VC—vibrocentrifuged concrete [15,17,26].

The initial comprehensive investigations into the physical and mechanical characteristics of centrifuged concrete were conducted during the 1950s and 1970s. The widespread practical use of this material for the construction of overhead power lines, which required new, more optimal and structurally advanced supports that could be manufactured on an industrial scale, caused the growing popularity of this topic among researchers. The studies yielded results that allowed for the formulation of applied theories and enhanced the understanding of the fundamental correlations, leading to a more precise prediction of the physical and mechanical characteristics of centrifuged concrete [76]. Table 2 and Figure 7, Figure 8 and Figure 9 show the main studies of the physical and mechanical properties of variatropic concretes.

As can be seen from the Table 2 and Figure 7, Figure 8 and Figure 9, when comparing variatropic concretes made via centrifugation and vibrocentrifugation, the latter demonstrate the best physical and mechanical characteristics (4–57% greater, depending on the property, section layer, and type of control).

In [27], the issue of the influence of the chemical activation of aggregates on the properties of lightweight, vibrocentrifuged, fiber-reinforced concrete was considered. Researchers found that using natural bischofite at a concentration of 6 g per 1 L of water to activate both fine and coarse concrete aggregates leads to optimal increases in strength and deformation characteristics. The authors reported a 16% increase in strength characteristics. The increase in compressive strain was 31%, while tensile strain increased by 21%. The elastic modulus increased to 9%. According to the authors, the results suggest a solution to the technological problem of activating inert concrete components by 20%, with low energy, resource, and labor requirements. As stated by the authors, the study involved the creation of an advanced lightweight, fiber-reinforced concrete and the proposal of a new technology aimed at achieving manufacturing savings by significantly reducing scrap and enhancing structural properties. By reducing the working sections of reinforced concrete elements made from this type of concrete, an economic efficiency effect of 25–27% can be achieved. The authors of [34] studied the influence of transverse spiral and longitudinal high-strength reinforcement on the physical and mechanical properties of centrifuged reinforced concrete elements with a ring cross-section. The authors analyzed a database of test results for almost 200 reinforced and more than 100 reference prototypes. The variable factors in the study were the coefficients of longitudinal (from 1.0% to 6.0%) and transverse (from 0.25% to 1.25%) reinforcement, as well as the pitch of transverse helical reinforcement (from 100 to 40 mm) and concrete strength (from 25 MPa to 60 MPa). Based on the results of the analytical study, experimental dependences of concrete strength coefficients, elastic moduli, and limits of longitudinal deformation of centrifuged concrete in reinforced concrete structures under short-term concentric compression were proposed. The main objective of study [11] was to assess how variatropy affects the strength changes in concrete structures subjected to humidification–drying cycles. Standardized and proprietary testing methods for concrete samples were used. It has been established through research that there is a correlation between the number of moistening and drying cycles and the strength characteristics of the samples, with a slight initial increase followed by a decrease. As stated by the authors, the strength characteristics of concrete produced using different technologies are predominantly compromised by an acidic environment. The strength characteristics of samples saturated in a neutral and alkaline environment exhibit nearly equal levels of deterioration, yet the neutral environment is considered to be more favorable. The findings revealed that vibrocentrifuged concrete exhibits a greater resistance to aggressive environmental conditions and wetting–drying cycles, in comparison to centrifuged and vibrated concrete. The drop in strength was up to 7% less, in comparison with centrifuged concrete and up to 17% less than in vibrated concrete. The study in [35] is devoted to an experimental study of work under load during the axial compression testing of ring-section elements made of centrifuged concrete with a fiberglass shell. In total, the authors tested 17 prototypes, which were divided into two groups, in each of which, the samples had 4 different lengths and included 13 straight and 4 conical columns. The authors assessed the influence of the initial eccentricity of the load application on the characteristics of the structure. A number of proposals have been developed to improve existing calculation methods to better match the calculated values to the experimental results. The study in [12] examined the issue of the resistance of variatropic concrete to cyclic and continuous sulfate corrosion. The authors’ findings indicated that vibrocentrifuged concrete exhibited the least amount of mass loss, at 17% less than centrifuged concrete and 37% less than vibrated concrete, when subjected to cyclic sulfate corrosion. The reduction in the cubic and prismatic strength of vibrocentrifuged concrete is minimal compared to centrifuged concrete, with reductions of 20% and 18%, respectively. In the case of vibrated concrete, there are even more pronounced decreases, with a respective reduction of 42% and 38% in cubic and prismatic strength. The corrosion resistance of concrete was evaluated by assessing its performance in a 5% sulfuric acid solution over a period of 180 days. It was found that vibrocentrifuged concrete exhibited the lowest corrosion rate and depth of damage. In [57], the authors studied the restraint effect in concrete composite columns of annular section with a steel shell under axial compression. In total, 18 prototypes of annular-section columns made of centrifuged concrete with a length of 450 mm; 15 annular-section cylinders of centrifuged concrete; and 15 prisms of vibrated concrete, with dimensions of 100 × 100 × 400 mm were manufactured. The cylinders were manufactured in a similar manner to the columns, but the steel shell was removed from them before testing. Based on the test results, it was concluded that the compressive strength of centrifuged concrete in a steel shell is 1.2–2.1 times higher than its vibrating counterpart, as well as also being 1.25 times higher than the calculated values. The experimental results were verified using parametric finite element analysis. According to the authors, centrifugation technology makes it possible to obtain effective composite elements from concrete with a high water–cement ratio. The local buckling of the steel shell in a composite column with an annular section is influenced by the ratio of the pipe diameter to its thickness and the depth of the concrete core. In [58], the authors consider the issue of studying the corrosion effect on the mechanical properties of centrifuged ring-section struts using three-dimensional finite element analysis. The influence of unevenly distributed corrosion products was modeled, taking into account deformations due to volumetric expansion over 75 years. Based on the results of the study, stress–strain curves in the corroded materials of the centrifuged piles were compiled and the adhesion and slip relationships between the corroded prestressed reinforcing bar and the concrete were presented. In order to estimate the degree of degradation in the mechanical properties of corroded pile materials, the authors introduced a set of equations. These equations were then compared to similar ones presented in earlier research. In accordance with the authors’ findings, the compressive strength of surface-corroded concrete showed a significant decline, once the corrosion rate had surpassed 12%. The decrease in bond strength exhibited an inverse exponential relationship with previous years, once the corrosion rate had surpassed 1.3%. The yield strength of the prestressed reinforcing bar demonstrated a linear decline as the degree of corrosion progressively increased, in this instance. The researchers in [59] examined the matter of assessing the efficacy of different chemical additives and their impact on the strength and deformability of reinforced centrifuged concrete components, when subjected to the adverse long-term combined influence of aggressive, salt-saturated groundwater and temperature fluctuations. The researchers experimented with 96 prototypes of centrifuged concrete. Multiple cycles of exposure to combined aggressive conditions were applied to the samples during testing. Extensive research has shown that the utilization of chemical additives, aimed at reducing the initial water-to-cement ratio (W/C), plays a crucial role in improving the mechanical attributes of centrifuged concrete applied in hot and arid climates, particularly in the presence of physical salt aggression. Superplasticizers have the greatest impact on reducing the initial water-to-cement ratio and, through a distinctive method of compacting concrete, contribute to the formation of an exceptionally uniform and dense concrete structure. The focus of the work in [13] revolves around evaluating the physical and mechanical characteristics, as well as the long-lasting nature of the variatropic structure of concrete when subjected to freezing and thawing cycles. Concrete specimens subjected to vibration, centrifugation, and vibrocentrifugation exhibit variations in weight loss and strength. Specifically, the weight loss percentages are 4.5%, 3%, and 2%, respectively, while the strength reductions are 15.0%, 13.5%, and 10%, respectively, when tested for frost resistance under similar conditions using the accelerated method after 15 cycles. As stated by the authors, centrifuged and, in particular, vibrocentrifuged variatropic concrete demonstrates an enhanced resistance and endurance to alternating freezing and thawing cycles when compared to vibrated concrete. The study conducted by the authors of [60] investigated the characteristics of centrifuged concrete treated with different chemical additives, subjected to prolonged exposure to saline-saturated groundwater and a cyclic temperature gradient. A total of 64 prototypes of centrifuged concrete were produced and evaluated. These prototypes underwent multi-cycle processing (75–120 cycles) under challenging environmental conditions. It has been confirmed that the temperature gradient and the presence of salts have a negative long-term multi-cycle impact on the compressive strength, Young’s modulus, and the durability of concrete. Nevertheless, the incorporation of chemical additives enhances the composition of centrifuged concrete, thereby exerting a noteworthy influence on its physical attributes, mechanical characteristics, and long-term sustainability.

In general, the phenomenon of variatropy, which occurs due to the use of a centrifugal method of compacting a concrete mixture, helps to improve the physical and mechanical characteristics of concrete and durability in comparison with traditional concrete produced using vibration technology. In addition, the combination of centrifugal and vibration compaction methods in one technology, that is, the use of so-called vibration centrifugation, helps to obtain an improved variatropic structure of concrete, which has better characteristics in comparison with variatropic centrifuged concrete. This structure, in comparison with the variatropic structure obtained via centrifugation, is characterized by a greater strength of the outer and middle layers of the annular section.

### 4.2. Features of the Microstructure of Variatropic Concretes

**Figure 10:**
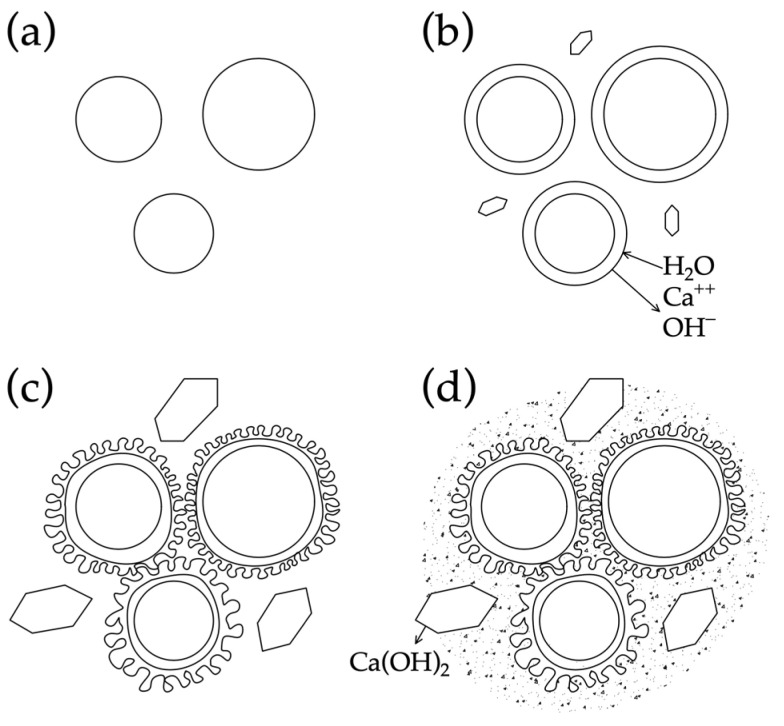
Scheme of the process of transformations in the structure of cement paste and hardened cement paste during cement hydration. (**a**) Cement grains in water—the initial period of hydration; (**b**) formation of a gel shell on cement grains—latent period of hydration; (**c**) secondary growth of the gel shell after osmotic destruction of the initial shell, the formation of wavy and columnar structures on the surface of the grains and in the pores of the hardened cement paste—the third period of hydration; and (**d**) compaction of the structure of the hardened cement paste during subsequent hydration of the cement.

The properties of centrifugally compacted concrete are determined by the variability of their structure at both the macro and micro levels. The structure of such concrete is formed due to the influence of centrifugal force, separately or together with vibration. During the centrifugation process, the concrete is compacted at the outer edge of the annular section of the centrifuged element, as well as the concentration of large filler particles in this zone, while, at the inner edge of the section, on the contrary, a less dense structure is observed, practically without large aggregates. Due to the significant compaction of concrete at the outer edge of the element, in variatropic concrete there is a significantly smaller number of open pores than in its vibrated analogues. As a result, the outer and middle layers exhibit a higher stability and density with fewer pores, in contrast to the outer layer, which often contributes to a decreased concrete resistance against aggressive influences. The layer contains structural pores that primarily serve as filtration channels. These channels increase in number and size as they approach the internal cavity of the annular element. Moreover, the hardened cement gel in this layer is characterized by numerous capillary pores [16]. Features of the formation of microstructure in centrifuged concrete are shown in Figure 10.

The indicated features of the microstructure and macrostructure of centrifuged concrete determine their main disadvantages. Consequently, solving the problems of increasing the strength and durability of centrifuged concrete and reinforced concrete elements is inextricably linked with the impact on their microstructure. The implementation of additives is a widely recognized and effective technique for enhancing corrosion resistance. One undeniable benefit of this approach is the ease of manufacturing and the affordability of most additives. Nevertheless, it is important to consider that the impact of additives in vibrated and centrifuged concrete may vary. The inclusion of surfactants, organic silicon liquids, and water-soluble resins does not prevent the development of directional porosity, such as migration pores and channels, within the inner layers of centrifuged products. This limitation hinders the desired enhancement of concrete durability and the prolonged lifespan of the resulting structures and products. This requires a specific evaluation of the efficiency of conventional additives when subjected to aggressive conditions in variatropic concrete. The most significant technical impact can be achieved through the impregnation of the solidified inner layer of concrete with a monomer, subsequently followed by its polymerization. The elimination of directional porosity, which is the main drawback of centrifuged and vibrocentrifuged concrete, was observed [77].

The Table 3 contains the influence of various factors on the formation of cement gel. 

As mentioned above, in the production of variatropic concrete, it is possible to experience either only centrifugal force or centrifugal compaction together with vibration. The latter approach to influencing the concrete mixture leads to the creation of vibrocentrifuged concrete. As a rule, the microstructure of vibrocentrifuged concrete is characterized by a denser crystalline intergrowth, which includes hydroaluminates, ettringite, and calcium hydroxides. Also, a higher amount of cement gel is observed in centrifuged and vibrocentrifuged concrete compared to vibrated concrete. It is also important to note the higher frost resistance of centrifuged and vibrocentrifuged concrete, which is due to the different nature of porosity and numerous reserve pores per unit volume, which are an additional degree of freedom for the distribution of hydrostatic pressure in the liquid during the formation of ice crystals under freezing conditions of water-saturated concrete. Compared with the traditional concrete structure, the advantage of the centrifugal concrete structure is that it protects the denser outer layer from moisture penetration. Furthermore, this effect, observed in Vibrocen triple melt concrete, allows for the controlling of the capillary porosity of the concrete, its properties, closure, and tightness, thereby strengthening the outer layer. The properties of the middle layer are closer to the values of the outer layer. With a double layer of protection against additional moisture, the capillaries are more reliably protected from internal stress and concrete damage during the freeze–thaw cycle. It is the structural and physical phenomena during the formation of these concretes that determine their great resistance and durability to alternating freeze–thaw cycles, which are also related to the dual effect of the outer layer protection and the inner porosity [13,69].

**Figure 11:**
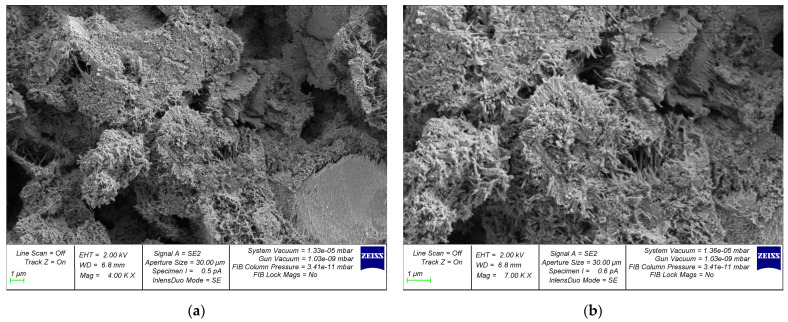
Photograph of centrifuged samples (**a**) at 4000× magnification and (**b**) at a magnification of 7000 times.

**Figure 12:**
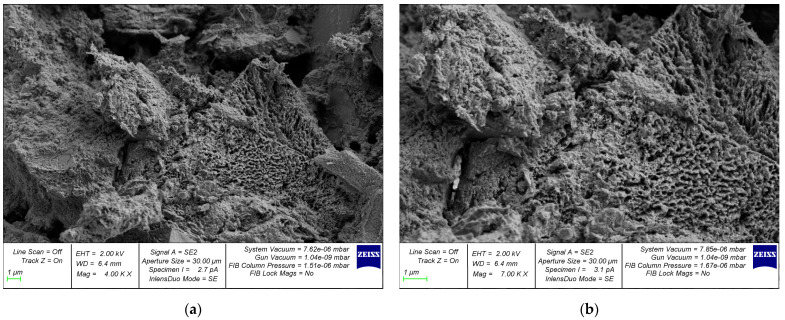
Photograph of vibrocentrifuged samples (**a**) at a magnification of 4000 times and (**b**) at a magnification of 7000 times.

During the initial phase of concrete structure development, the mixing water creates a network of interconnected capillaries and large pores within the concrete, distributed in a disordered manner throughout the composite’s entire volume. Furthermore, with the continuation of cement hydration, there is a reduction in both total and capillary porosity of the hardened cement paste. The explanation for this can be found in the greater volume of cement hydration products and the gaps between the newly formed crystals. These gaps are approximately 2.2 times larger than the volume of non-hydrated cement. In this way, the system of interconnected pores is effectively divided into discrete units by the cement gel. The composition of the solidified cement paste contributes to the reduced permeability of concrete, with the primary factor being the initial water–cement ratio. The macrostructure of concrete produced through the utilization of centrifugal compaction technology exhibits irregularities. In accordance with conventional categorization, it can be divided into the following three layers: internal, middle, and external [91]. The study in [69] is devoted to the investigation of the dependence of the processes of formation of cement gel and methods of the compaction of cement systems. The authors acquired novel insights and formulated theoretical concepts pertaining to the cement gel formation processes in three technologies, as follows: the vibration, centrifugation, and vibrocentrifugation of concrete. They identified key distinctions in gel formation, outlined the principal physical and chemical processes involved, and highlighted the substantial impact of technology on the gel formation process. The study assessed the impact of indirect characteristics, specifically the processes of cement gel formation, rheological properties of concrete mixtures, water extraction processes, and the liquid-to-solid phase ratio in the mixture. Extensive research has been conducted on the formation process of cement gel in centrifugally compacted cement systems. Graphical representations have been developed to elucidate the interaction mechanism based on the principle of “composition—rheological characteristics—structure—properties of concrete”. The achieved results’ quantitative aspect was demonstrated through the increases in indicators observed in the centrifuged and, specifically, vibrocentrifuged samples, when compared to the vibrated samples. When considering strength indicators, it was observed that the vibrocentrifuged samples displayed a varying increase, ranging from 22% to 32%, depending on the specific strength type. Figure 11 and Figure 12 show a clear difference in the microstructure of centrifuged and vibrocentrifuged concrete samples [69].

SEM photographs clearly demonstrate the differences in the microstructure of variatropic concretes produced using different technologies. The centrifuged concrete sample (Figure 11a) has noticeable pores and voids compared to the vibrocentrifuged concrete sample (Figure 12a). This observation confirms the stronger and more organized structure of vibrocentrifuged variatropic concrete, in comparison with centrifuged concrete, which is supported by the given quantitative indicators of the physical and mechanical properties of concrete [69]. The higher magnification photographs (Figure 11b and Figure 12b) demonstrate a smaller number and size of filtration channels in vibrocentrifuged concrete, compared to centrifuged concrete, which is in good agreement with [23,24]. Thus, it is obvious that the microstructure of variatropic concrete has its own characteristics in comparison with traditional types of concrete. Of course, the weak point of variatropic concrete is the so-called inner layer, which has the lowest cross-sectional thickness and density, in comparison with the middle and outer layers. In this regard, to eliminate the formation of migration pores and channels in the internal layers of centrifuged products, as well as ensuring the required increase in the durability of concrete and the service life of the products and structures made from it, it is necessary to use special types of additives and impregnations that work effectively under conditions of aggressive influence on variatropic concrete. The most significant technical impact can be achieved by saturating the solidified internal concrete layer with a monomer, subsequently followed by polymerization. The primary drawback of variatropic concrete—directional porosity—is effectively eliminated. When describing the characteristics of the microstructure, its relationship with the characteristics of concrete produced by the centrifugal method should be taken into account. The most important parameter of the microstructure is the formation of a dense packing of particles with variable density across the cross-sectional thickness. At the same time, at the macro level, this is expressed as the compact filling of smaller fractions between the empty spaces between large grains, while, at the micro level, it looks like the formation of clusters of hydrogenated cement grains, uniformly enveloping the inert components. In this case, the formation of layers of variatropic microstructures of concrete occurs smoothly and inextricably. Subject to a rational choice of recipe and technological factors, such as rotation speed, rotation frequency, as well as the composition of concrete, the microstructure of concrete becomes as efficient and smooth as possible, with the characteristics of all layers increasing to the greatest extent. Then, the characteristics of concrete, both overall and in layers, will be as effective as possible. Thus, a direct relationship between the microstructure and characteristics of centrifugal concrete is confirmed.

## 5. Structures Using Variatropic Concrete

When considering variatropic concrete as a material for building structures, one cannot fail to take into account the specifics of the process of their manufacture. As mentioned above, a feature of the structure of variatropic concrete is its variability in the cross-section, depending on the distance from the central axis of the element. Achieving such a material structure is determined by the manufacturing process of the structure. Today, structures made of variatropic concrete are manufactured using centrifugation, while ensuring the required speed of the process and its duration. Structures made in this way have a ring cross-section, with the highest strength and density of concrete on the outer face and the lowest values of these parameters on the inner face of the ring [92]. Initially, centrifugation was used to make structures for reinforced concrete pipes and power line supports, the need for the mass production of which led to the increase in the use of centrifuged concrete in the mid-twentieth century. However, centrifuged concrete finds its application not only in these structures, but also in a variety of high-rise tower structures, telecommunications mast structures, and the like. For these structures, it is very important to ensure the simplicity of their calculation, design, and installation. The main competition for centrifuged concrete in this area is steel structures with a lattice cross-section [93]. Speaking about reinforced concrete structures, it can be noted that with a relatively small height, tower structures can be made with a solid cross-section; however, when constructing structures of great height, centrifuged elements of an annular cross-section are used, which have a higher load-bearing capacity due to the technological features of their production [94]. Today, centrifuged annular elements are used not only as pipe structures and tower elements, but also as short columns in transportation structures such as runway extensions, airports, islands, and overpasses. Also, the centrifuged elements of annular cross-sections are actively used as structures for large tanks (hoppers), wind generator masts, and chimneys. Today, glass and carbon fiber reinforcement are actively used in the designs of pipes and tanks, which can significantly increase the corrosion resistance of the structure [61].

The high durability of structures made of variatropic concrete is due to the high density of the outer surface of the element, which, thanks to this factor, has minimal water absorption due to the small number of open pores. Therefore, when operating such structures outdoors, there is no significant decrease in their strength over time [36]. It is also important to note that, unlike steel structures, reinforced concrete elements do not require constant maintenance in the form of repainting and corrosion protection. At the same time, reinforced concrete structures have architectural appeal and fit harmoniously into the urban environment.

For the construction of tower structures of great height, on the order of tens and hundreds of meters, the issue of optimizing the cross-section of their main elements becomes relevant, which would improve their technical and economic efficiency. However, the methodology for selecting an effective cross-section is very limited in its results and researchers are increasingly using a potentially more effective approach associated with the creation of new structural forms. As a rule, in this case, the objects of research are combined with structural systems using prestressed struts. At the same time, the use of prestressed elements makes it possible to reduce the bending moments of such columns and their design length and, therefore, increases their load-bearing capacity [64]. The study in [65] is devoted to the issue of studying the operation of communication mast structures, consisting of a combined system of prestressed columns and a core made of the centrifuged concrete of an annular section. According to the authors, their proposed structure will make it possible to achieve a significantly greater efficiency of such masts. The authors analyzed the behavior of a mast structure made of prestressed columns with a core of centrifuged concrete of an annular section, taking into account the geometric and physical nonlinearity of its elements. The authors also assessed the strength and stability of the centrifuged concrete core of such a combined system. The authors analyzed prestressing for internal stresses and the displacement of the reinforced concrete core. The analysis results were compared with the characteristics of traditional tower-mast structures. The authors concluded that the efficiency of the new structural system increased by 2.5 times compared to a classic mast and by 5 times compared to a standard tower system.

The study in [38] focuses on the long-term behavior of a series of heavily loaded spin-concrete pier specimens, prestressed with carbon fiber reinforced polymer reinforcement, which have been subjected to open four-point bending creep tests since 1996. The authors studied cylindrical beams with a span of 2 m. Also, as part of this work, the authors examined five samples of thin-walled pillars (diameter 100 mm, wall thickness 25–27 mm). All samples were prepared using the pre-tensioning and rotation method. Initially, two support column specimens were tested in quasi-static four-point bending, to determine the moment of short-term failure and to simulate the behavior under short-term bending. The three pole specimens were then loaded at different creep bending moments; while the specimen with the lowest load was initially uncracked, the second specimen was loaded at a 50% short-term creep bending moment and showed cracking immediately after application of the load. The most-loaded support specimen withstood a bending moment equal to 72% of the short-term bending failure moment for 16.5 years, before failing in July 2013 due to reinforcement failure, resulting in local failure of the high-performance centrifuged concrete. In addition, long-term monitoring of creep tests showed a limited, time- and temperature-dependent increase in deflections over the years, mainly due to concrete creep. The concrete creep model made it possible to calculate the long-term bending curvature with sufficient accuracy. In addition, the support samples exhibited time-sustained crack structures and minimal reinforcement slippage, relative to the support ends for the two lower load levels.

The study in [66] is devoted to the issue of the compressive strength of the concrete pipes of the annular section with a fiber–plastic hybrid shell. The experimental results were compared with the results of calculations carried out using the finite element method. The authors claim that the compressive strength of the hybrid shell increased by 14% when comparing the longitudinal compressive strength of a pre-tensioned, high-strength concrete pile and a concrete-filled pipe.

The authors of [14] addressed the matter of modeling and calculating variatropic concrete structures in centrifuged reinforced concrete columns. The issue of compressing hollow columns constructed with variatropic concrete, which is both homogeneous and non-uniform along the annular section, reinforced with steel bars at various levels of load eccentricity was examined. The numerical solution was employed within the ANSYS environment to resolve the issue concerning a vertically oriented column that was rigidly fixed at the lower edge and subject to eccentric loading at the upper edge. Three types of eccentricity *e*/*r* = 0, 0.16, and 0.32 (0, 24 mm, and 48 mm, respectively) were examined. The obtained results of the solution included stress and strain fields, as well as a visual representation of crack development within a spatial framework. The findings indicate that the homogeneous column outperforms the variable column in terms of load-bearing capacity by 3.6% in central compression. The variatropic column exhibits a higher load-bearing capacity (5.5% and 6.2% greater) compared to a homogeneous one, at eccentricity values of *e*/*r* = 0.16 and 0.32, while also demonstrating a superior resistance against crack formation. The work in [67] assessed environmental and economic performance by analyzing the resource use and installation processes of prestressed high-strength piles. The authors propose a process modeling method that breaks parts down to the work task level, based on energy consumption and resource costs, and a modeling method that calculates the environmental–economic performance of the process and resources of a high-strength prestressed pile (e.g., equipment). The authors presented a quantitative comparison of duration, costs, and emissions derived from simulations, estimates based on standard construction performance, and detailed unit costs. The authors claim that the proposed methods effectively predict the duration, cost, and carbon emissions occurring in real-world conditions during the construction planning phase.

The study in [62] examines the impact of the production processes and use of concrete in the structures of buildings and infrastructure on global warming. The authors calculated the global warming potential for prestressed reinforced concrete piles. Based on the results obtained, the impact of precast concrete structures on global warming was assessed. The authors state that despite the high global warming potential of on-site concrete fabrication processes, precast concrete fabrication processes have a significantly lower global warming impact.

The authors of [68] explored the use of locally produced steel fibers and commercially available polypropylene fibers to develop an improved concrete mix for use in the production of full-scale spin-cast concrete pipes. The authors used the following two types of steel fibers: straight and bundled. The authors used different dosages of steel (20, 30, 40, and 50 kg/m^3^) and polypropylene fibers (5, 10, 15, and 20 kg/m^3^). The mechanical properties of the resulting compositions were studied both in the fresh state and after hardening. The authors produced full-size prototypes in the form of centrifuged pipes with an internal diameter of 450 mm. The prototypes were tested for bending using a three-point load application scheme. According to the authors, the strength of each composition was practically independent of the dosage of fibers. The tensile strength of all compositions was higher than that of the control mixture. An increase in strength of 24% was noted for a composition containing bundles of steel fibers in an amount of 50 kg/m^3^. On average, the flexural strength of all mixtures was 28% higher compared to the composition without fibers. The authors of [15] examine the matter of establishing universal approaches to investigating the integral and differential attributes of concrete in their work. The selected article discusses the methods and instrumental apparatus used for experimental studies on centrifuged and vibrocentrifuged concrete products with annular cross-sections. These methods allowed for an evaluation of the actual variatropy of the structure and verification of the research direction. The authors put forward a novel approach to conduct experimental investigations on the variatropy of vibrated, centrifuged, and vibrocentrifuged concrete sections. This method aims to determine the integral and differential strength and deformation characteristics, as well as deformation diagrams. It was proven that with vibratory centrifugation, it becomes possible to obtain concrete with an improved structure and with higher characteristics than with centrifugation and vibration. The examination of centrifuged and vibrocentrifuged concrete under compression and tension in experimental studies indicated that the outer layer of concrete exhibits the most favorable characteristics, whereas the inner layer displays the least favorable characteristics. The authors experimentally confirmed the rationale for the three-layer model of the variatropic structure of centrifuged and vibrocentrifuged concrete. During the study, a differentiation in the characteristics of the layers of variatropic concrete was obtained, whereby the concrete of the outer layers has the greatest strength, elastic modulus, and the lowest deformability; the concrete of the internal layers has the lowest strength, elastic modulus, and highest deformability; and the concrete of the middle layers has average characteristics. The deformation diagrams of centrifuged and vibrocentrifuged concrete were also differentiated by layer, confirming the variable structure of such concrete. Of these, the deformation diagrams for the outer layer of concrete were the greatest in terms of strength; the lowest strength was observed for the inner layer of concrete; and the average indicators were the deformation diagrams for the middle layer of concrete.

When describing the characteristics of structures made of variable-granular concrete, it is important to always take into account the characteristics of the mechanical properties of the concrete itself, since their values differ from the commensurate characteristics of vibrated plain concrete. This is due to the variability of the structure, that is, changes in the parameters of the density and strength of centrifugally compacted concrete along the thickness of the section. At the same time, the structure, which is made of variable-granular concrete, has its advantages and features. For example, the outer layer of the structure is more dense and durable, which allows it to interact with the environment more effectively, and the inner layer is the least dense and durable, but, nevertheless, it does not actively participate in the formation of the operational reliability of the structure. This expresses the interdependence of the characteristics of structures made of variable-grained concrete and the characteristics of variable-grained concrete.

## 6. Discussion

The analytical review carried out here made it possible to identify a number of important new provisions on the technology, structure formation, and properties of variatropic concretes, allowing us to systematize the existing knowledge in these areas and to create an important basis for future scientific and applied research.

In particular, a systematic review of the types of centrifugal installations for producing variatropic concrete is noted. At one time, various authors, such as [15,19,22,23,25,33,56,64,95,96], dealt with the issues of compiling a knowledge system about variatropic concretes, but, at the same time, their description of equipment and accessories was not interconnected in one study on the issues of the structure formation and properties of variatropic concretes.

Next, attention should be paid to the theoretical justification for the formation of the variatropic structure of centrifuged concrete. At one time, Akhverdov I.N. [23] and Gershberg O.A. [22] studied this issue, but, at the present stage, there are some new models for the formation of variatropic structures associated with new possibilities in the formulation, technology, and design of such elements. In particular, an example could be the modification or fiber reinforcement, as well as the issues of the durability of such materials in reinforced concrete structures, which ultimately leads to the improvement of life cycle management processes of buildings and structures, obtaining hidden reserves that allow for the more rational use of the stock of reinforced concrete variatropic structures.

An important aspect is the study of the microstructure of variatropic concretes at the level of processes, including the gelation or hydration of cement. This is a very important point, which allows us to interconnect technology, formulation, structure formation, and properties of variatropic concretes into a single system of knowledge. The issue of using real structures is very important in terms of the applicability of the results of the analytical review in practical construction and production. This is also carried out for the first time, with reference to theoretical aspects.

After analyzing all the relationships between the composition, structure, technology, and properties of centrifugal RC, selected successful examples of full-scale experimental data and practical applications of structures made from centrifugal RC should be given. It should be noted here that centrifugal RC is used in various industries and fields. For example, in the energy construction industry, RC is used to make power transmission poles, as well as railroad catenary poles. In the infrastructure construction industry, centrifugal RC is used to make culverts. In industrial and civil construction, centrifugal RC is used as pillars and columns. At the same time, various types of studies with full-scale experiments [14,15,16,22,23,25,34,37,51,60] have shown that the life cycle of such real structures significantly exceeds the life cycle of structures made of simple vibrated concrete, which is confirmed by data from various authors.

Thus, the conducted analytical review represents a theoretical and practical basis of new systematized knowledge, with an understanding of the fundamental principle of technology–composition–structure–properties regarding variatropic concrete, which ultimately leads to the creation of new improved reinforced concrete elements with a variatropic concrete structure with increased durability and performance reliability, using all the hidden reserves of such promising variatropic structures in practice.

## 7. Conclusions

This article provides a comprehensive literature review on the recent research conducted in the field of variatropic concretes. The main structures created using centrifugation technology are considered. An analysis of the basic physical and mechanical properties of variatropic concretes is carried out and the features of their microstructures are considered. A theoretical justification for the formation of the structure of variatropic materials is presented. Based on an examination of the presented review, the following primary findings can be inferred:

(1) Variatropic concrete is a promising building material due to the peculiarities of changing its physical and mechanical properties within the cross-section.
(2) The phenomenon of variatropy, which occurs due to the use of a centrifugal method of compacting a concrete mixture, helps to improve the physical and mechanical characteristics of concrete in comparison with traditional concrete, produced using vibration technology. Variatropic concrete has improvevd mechanical characteristics compared to traditional concrete, on average by up to 45%. The combination of centrifugal and vibration compaction methods in one technology, that is, the use of so-called vibrocentrifugation, helps to obtain an improved variatropic concrete structure that has better characteristics in comparison with variatropic centrifuged concrete, on average by up to 20%. This structure, in comparison with the variatropic structure obtained via centrifugation, is characterized by a greater strength of the outer and middle layers of the annular section.
(3) The advantages of the variatropic structures of centrifuged concrete due to the protection of a denser outer layer from moisture penetration, compared to a conventional concrete structure are noted and explained. This effect is observed to a greater extent in variatropic vibrocentrifuged concrete, which allows for the controlling of the capillary porosity of concrete and its nature, as well as strengthening the outer layer and pulling the values of the middle layer to the values of the outer one, resulting in a double layer of protection from additional moisture; the capillaries are even more reliable when protected from internal stresses and destruction of concrete when exposed to aggressive factors.
(4) The durability of concrete with a variatropic structure, characterized by the porosity of the outer surface, its water absorption, resistance to the penetration of chloride ions, alternating cycles of freezing and thawing, as well as cycles of moistening and drying, is, all other things being equal, higher than that of concrete of a conventional structure. The durability of variatropic concrete is improved by up to 30% compared to conventional concrete.
(5) Vibration centrifugation technology is proposed for implementation in structures operating in special conditions with an increased risk of sulfate corrosion in concrete and reinforced concrete.
(6) The first studies on the topic of variatropic concrete were aimed at obtaining new data on the influence of the ratios of mixture components and centrifugation parameters on the strength and physical–mechanical properties of the final material. Modern research uses new ideas and technologies and needs further, more detailed study.

The conducted review study presented in this article, against the background of the almost complete absence of review articles on variatropic and centrifuged concrete, partially solves the problem of a certain scientific deficit in this area, through an integrated approach to the analysis of research and its systematization.

## Figures and Tables

**Table 1:** Main directions of research into the properties of variatropic concretes.

Reference	Manufacturing Technology	Main Focus of the Study
[26,27]	Centrifugation Vibration centrifugation	Impact of component activation
[16,19,34,35,36,37,38,39,40,41,42,43,44,45,46,47,48,49,50,51,52,53,54,55,56]	Centrifugation	Mechanical properties
[11,13]	Centrifugation Vibration centrifugation	Effect of freeze–thaw cycles
[12]	Centrifugation Vibration centrifugation	Effect of exposure to sulfate attack
[57]	Centrifugation	Deterrence effect research
[58]	Centrifugation	Effect of corrosion
[59,60]	Centrifugation	Influence of salt attack and temperature factors
[61,62,63]	Centrifugation	Effect of fiber reinforcement
[64]	Centrifugation	Work of structures
[65]	Centrifugation	Creep research
[66]	Centrifugation	Modeling and calculation
[14]	Centrifugation	Economic and environmental impact
[67]	Centrifugation	Impact on global warming potential
[15,17,68,69]	Centrifugation Vibration centrifugation	Mechanical properties
[70,71]	Centrifugation	Microstructure analysis methods
[18]	Centrifugation Vibration centrifugation	Effect of chloride attack
[72]	Centrifugation Vibration centrifugation	Influence of nano modifiers

**Table 2:** Studies of the physical and mechanical properties of variatropic concretes.

Reference	Type of Variatropic Concrete under Study	Modulus of Elasticity, GPa	Compressive Strength, MPa	Axial Tensile Strength, MPa
[26]	Centrifuged Vibrocentrifuged	35.9 40.1	54.9 60.3	4.3 4.8
[34]	Centrifuged	–	30.0–50.0	–
[12]	Centrifuged Vibrocentrifuged	–	47.3 50.8	–
[57]	Centrifuged	30	20.0	1.5
[58]	Centrifuged	–	53.0	–
[59]	Centrifuged	–	49.1	–
[13]	Centrifuged Vibrocentrifuged	–	51.0 54.1	–
[60]	Centrifuged	40.8	57.2	–
[66]	Centrifuged	43.5	38.7	3.87
[62]	Centrifuged	–	32.6	3.21
[15]	Centrifuged Vibrocentrifuged	39.9 42.9	62.5 70.7	4.6 5.3
[16]	Centrifuged Vibrocentrifuged	34.3 38.8	43.4 68.2	–
[17]	Centrifuged Vibrocentrifuged	32.8 33.9	45.7 49.9	5.9 6.4
[37]	Vibrocentrifuged	28.2	38.2	2.7
[69]	Centrifuged Vibrocentrifuged	–	43.1 44.7	–

**Table 3:** Influence of various factors on the formation of cement gel.

Reference	Type of Factor Taken into Account in the Work	Name of the Influencing Factor	The Influence of the Factor under Consideration on the Formation of Cement Gel and the Microstructure of Hardened Cement Paste
[78]	Prescription (use of nanomodifying additive)	nano-SiO_2_	The addition of nanosilica helps to increase the degree of hydration of the cement paste and increase the active formation of the calcium silicate hydrates (CSH) gel, as well as to reduce the overall porosity of the hardened cement paste
[79,80]	Prescription (use of additive as a replacement for part of the cement)	bottom ash from power plants	Cement pastes with added bottom ash contain more CSH than cement pastes without the additive.
[81]	Prescription (use of additive as a replacement for part of the cement)	low calcium fly ash	The loose and porous microstructure of fly ash results in higher water consumption, which reduces the fluidity of cement-based pastes. The main hydration products of cement-based materials mixed with LCFA were AFt, CSH gel, and Ca(OH)_2_
[82,83]	Prescription (use of additive as a replacement for part of the cement)	belite cement and fly ash	Hydration of belite cement from fly ash promotes the formation of CSH, ettringite, and calcium hydroxide gel, thereby significantly increasing long-term strength and also reducing the porosity of the hardened cement paste
[84]	Prescription (use of nanomodifying additive)	carbon nanofibers	Carbon nanofibers fill nanopores and connect grains of CSH, while nanofibers influence the probability distribution function of the local packing density, causing a shift towards higher values
[73,85]	Prescription	radioisolators Bi_2_O_3_ and ZrO_2_	Radiocontrast agents help increase the average length of the silicate chain and the degree of aluminum substitution in the CSH gel
[85,86]	(additive use)	titanium nanoparticles	TiO_2_ nanoparticles promote compaction of cement paste and, as a result, increase strength
[87,88]	Prescription (use of nanomodifying additive)	carbon nanotubes, nanosilica	Nanosilica absorbs on the surface of carbon nanotubes and promotes the formation of hydration products on their surface, which improves adhesion between carbon nanotubes and the cement matrix
[85,88,89]	Prescription (use of nanomodifying additive)	carbon nanotubes	Carbon nanotubes form strong interfacial bonds with cementitious matrices and also increase the proportion of high-density calcium silicate hydrate (HD-CSH) gel compared to low-density CSH gel
[90]	Prescription (use of nanomodifying additive)	calcium sulfoaluminate cement and gypsum	It is noted that the gel transition time decreases with increasing amounts of calcium sulfoaluminate and gypsum additions
[91]	Prescription (supplement use)	coal gangue and slag powder	The resulting three types of binder gels are characterized by a dense structure and high strength

## Data Availability

Not applicable.
